# Identifying the Gaps in Practice for Combating Lead in Drinking Water in Hong Kong

**DOI:** 10.3390/ijerph13100970

**Published:** 2016-09-30

**Authors:** Wai Ling Lee, Jie Jia, Yani Bao

**Affiliations:** Department of Building Services Engineering, The Hong Kong Polytechnic University, Hong Kong, China; jiajie.shsf@gmail.com (J.J.); jenny.bao@connect.polyu.hk (Y.B.)

**Keywords:** lead in water, engineering aspects, quality control system, regulations and ordinances, allowable lead contents, testing and commissioning

## Abstract

Excessive lead has been found in drinking water in Hong Kong in tests carried out in 2015. Investigations have identified that the problem in public rental housing estates was caused by the problematic solders used in the plumbing, and recommendations on enhancing the quality control system and strengthening the relevant water quality standards have been proposed. The cause for the same problem happening in other premises where soldering has not been adopted for water pipe connections is left unidentified. Considering the unidentified cause and the recommendations made, this study aims to identify the gaps in practice followed in Hong Kong for safeguarding the water quality of new installations. A holistic review of governing ordinances and regulations, products and materials used and the testing and commissioning requirements adopted in Hong Kong and elsewhere in the world were conducted. Based on international practices and parametric analysis, it was found that there are gaps in practices followed in Hong Kong, which are directly and indirectly leading to the lead-in-water crisis. Recommendations for improvement in the quality control system, and the water quality standards including the allowable lead content and leaching limit for products and materials and the testing and commissioning requirements on plumbing installations have been made. The review and the identified gaps would become useful reference for countries in strengthening their relevant water quality standards.

## 1. Introduction

The quality of water that comes out of the tap is affected by both the distribution system that starts from the water treatment plants and the plumbing installation inside buildings. In the entire water supply system, water can become exposed to a range of chemicals, metals and bacteria. Amongst them, the existence of metallic lead can have an acute health impact in the long term [1,2,3] and thus is receiving much attention. 

Compared to other countries, Hong Kong has very strict guidelines about how much lead can be in tap water. It adheres to the World Health Organisation (WHO) standard of 10 µg/L [4] which is less than in the United States (US) [5] or in mainland China [6] where the standards are 15 µg/L and 50 µg/L, respectively.

In 2015, samples of potable water from public rental housing (PRH) estates in Hong Kong were found to contain excessive levels of lead. Since such discoveries, water tests using different sampling protocols have been carried out almost everywhere in Hong Kong, including kindergartens, day care centres, schools, private and public housing estates and hospitals. Unfortunately, many have shown lead concentrations more than three times higher than the WHO’s recommended level [7]. This bad news caused widespread concern about a lead-in-water crisis in the city. The residents were panicking over the uncertainty if the tap water was safe to drink. 

The quality of water on the distribution side in Hong Kong is the responsibility of the Water Supplies Department (WSD) which actions has always been good because water quality, including the lead concentration, has been closely monitored to ensure conforming to WHO guidelines [8]. As the majority of Hong Kong people are living in high-rise buildings, a small amount of residual chlorine is also maintained in the water to keep it free from bacterial infection during its long journey in the distribution system. The concern is therefore confined to plumbing installations inside buildings where copper pipes and copper and copper alloy fittings are often used. The WSD therefore jointed hands with the Housing Department (HA), the developer for PRH estates in Hong Kong, to lead a review committee (the Committee) on the excess lead-in-water problem in PRH developments.

The Committee’s investigation focused on housing estates completed during and after 2005 where soldering was adopted for water pipe connections. Systematic water sampling tests were conducted at 86 PRH estates, involving 83 PRH developments. A total of 4821 water samples were taken. The report released on 8 January 2016 [9] confirmed that excessive levels of lead were found at 11 PRH developments (approximately 29,077 units), and the problem was caused by the problematic solders used. In connection, WSD and HA have taken a series of immediate measures including the provision of water wagons/tanks, supply of bottled water, the installation of temporary water points by connecting pipes from the roof-top tank to each floor, as well as free installation of water filters (including replacement filter cartridges for two years after installation) for the affected domestic households. Further recommendations were to enhance the work quality of the plumbing contractors/sub-contractors including supervision and on-site monitoring, soldering materials used and training.

Besides the task force, the Chief Executive of the Hong Kong SAR Government also appointed a commission of inquiry into the excess lead-in-drinking water problem in the territory (the Commission) aiming to ascertain the causes for the problem and to make recommendations for improvements.

The Commission’s investigation report was released on 31 May 2016 [10]. A similar cause was identified for the excessive lead-in-water in PRH estates but not for other premises. This has highlighted the inadequacy of the existing legislative framework in safe-guarding the quality of drinking water in Hong Kong. The recommendations made were mainly on measures to enhance the quality control system including the setting up of an international and an independent panel on water safety, establishing the Hong Kong drinking water standard, and legislating the role and responsibilities of different parties responsible for design, construction and maintenance of plumbing system. 

Both the Committee and the Commission’s recommendations are targeting to safeguard the water quality of new building developments. However, there are no details on how the recommendations can be practically implemented on the engineering aspects. 

Reference was therefore made to relevant research works. For the excess lead-in-water problem, recent research works have been focused on assessing the associated health risk [1,2,11,12,13], developing new filtration technologies [14], computational modelling of different sampling approaches [15], and the corrosion potentials with different water chemistry [16,17,18,19], materials used [20,21,22,23] and disinfection practices [24,25,26,27,28,29,30]. On engineering aspects, many reference guidelines and products/materials standards for ensuring water quality have been published by different authorities worldwide and Hong Kong is no exception. Research works have also been done on evaluating the influences of different sampling methods on the water test results [31,32,33,34,35], optimizing the operational and water quality management strategies [36], and establishing a performance improvement framework [37]. However, there are virtually no single study that has focused on the whole spectrum of engineering aspects to ensure provision of safe drinking water.

## 2. Methods

To identify the inadequacies in the engineering aspects, in this study, a holistic review of the various project stages of a plumbing installation, involving the governing ordinances and regulations, the products and materials used and the testing and commissioning requirements, will be conducted. The main objectives are to identify inadequacies of practices followed in Hong Kong. 

Reference is made to trends and experiences in Hong Kong and outside, to the extent available, including other trades where applicable and available. Amongst overseas practices, through content and bibliometric analysis, the US and Canada no doubt have a representative role [38]. Whilst Australia and New Zealand are at the onset of research on the problem of lead in drinking water [39], the United Kingdom (UK) is the first country to have set up engineering standards for addressing health and safety issues in this context [40]. Most UK practices are often followed by Hong Kong because of the colonial history.

### 2.1. Governing Ordinances and Regulations

The following reviews the primary instruments governing the provision of safe drinking water in various countries and jurisdictions, aiming to identify regulatory controls over safe drinking water in buildings and the parties/persons involved to ensure compliance.

#### 2.1.1. International Practices

In the US, the Safe Drinking Water Act (SDWA) is the main federal law that ensures safe drinking water [41]. Under SDWA, the Environmental Protection Agency (EPA) sets standards and guidelines for drinking water quality and oversees different states, localities and water suppliers who have to follow and implement those standards.

Canada, Australia and New Zealand also adopt the SDWA as legislative protection for ensuring drinking water supplies are safe. In Canada, the enforcement responsibility is shared between the provincial, territorial, federal and municipal governments. The day-to-day responsibility of providing safe drinking water to the public generally rests with the provinces and territories, while municipalities usually oversee the day to day operations of the treatment facilities [42]. In Australia, the Department of Health and Human Services sets Australian Drinking Water Guidelines [43] and enforces the SDWA regulations. Whilst in New Zealand, the Act is administered by the Ministry of Health which also sets the guidelines for drinking-water quality management for New Zealand [44]. The UK adopts the Water Industry Act to set Water Supply (Water Quality) [45]. The enforcement authority is the Secretary of State for the Environment, Transport and the Regions.

To ensure that plumbing installations are in compliance with SDWA and other relevant regulations, only licensed plumbers (LP) are used for installation and repair works in all the above countries. LPs normally have years of training and/or experience. They need to be registered according to the relevant ordinances and employed by either the building client (or his representative) or the installation contractor. They are licensed to construct, install, maintain, alter, repair or remove plumbing installations.

Other than licensed plumbers, little information is available in the literature on the parties/persons involved in plumbing installations.

A comparison of the enforcing authority, regulatory hierarchy and relevant standards and guidelines are summarized in Table 1. It can be seen that guidance manuals and standards for controlling corrosion and excessive lead in drinking water are available for all of these countries.

#### 2.1.2. Hong Kong Practice

Hong Kong is no different from other countries. The primary instruments governing the provision of safe drinking water are the Waterworks Ordinance (Cap102) and the sub-legislature Waterworks Regulations (Cap102A) under the Water Authority delegated to WSD for enforcement. WSD is also responsible for setting standards but they largely make reference to British Standards [46]. A handbook on plumbing installations for buildings specific to Hong Kong is also available [47]. However, the handbook focuses on Hong Kong waterworks requirements in respect of policies, and water supply application procedures. Guidance manuals and standards for controlling drinking water quality in the local context is lacking.

For new and major renovation projects, the current Hong Kong building legislation system requires an Authorized Persons (AP) who should be an architect, registered according to the Buildings Ordinance and employed by the building client, to take overall responsibility to ensure building works are in compliance with the relevant Ordinances and Regulations. They need to certify the use of WSD approved pipes and fittings and proper completion of plumbing works [47]. But as most APs do not have the background to understand plumbing installations, their role, as revealed from a survey, is normally restricted to confirm “the correctness of the water meter positions” as indicated by the LP [48]. As for the registered professional engineers (mainly building services engineers), there is no specific role prescribed in the legislation system. Submissions of plumbing system design, materials used and installation details are the responsibility of the LPs and approval of submissions are by the WSD. Thus Hong Kong also relies on the use of LPs to ensure plumbing installations are in compliance with Waterworks and related Regulations. 

According to a survey of Hong Kong practices, plumbing installation works are often included under the Main Contract [48]. The plumbing installation contractors (PIC) are therefore employed by the main contractor as a domestic contractor to execute the installation works. The main contractors are typically listed in different classes according to their establishments, liquidity, track records, etc. to bid for projects of different scales and levels of complications but there is no registered list for the PICs. Thus the establishments of different PICs differ largely from each other. They may consist of only one LP for preparing and signing all submissions made to the WSD. As such, the actual installation work is carried out by semi-skilled or skilled plumbers but not a LP. 

The LPs are considered as one type of Technical Competent Person (TCP) working in the construction industry [49]. TCPs are classified into five grades (T1–T5). TCPs T4 and T5 carry out engineering safety supervision to ensure compliance with design assumption and requirements. TCPs T1–T3 carry out routine safety supervision to ascertain compliance with approved, accepted or submitted method statements and precautionary and protective measures. The LPs on their own are not classified into different grades. There is only one grade of LP. They receive only vocational education with a minimum of five years working experience [50], which is the qualifying requirement of a TCP T3. 

### 2.2. Products and Materials

Copper pipes have impurities such as lead. Lead is also frequently added to copper and copper alloy fittings to increase their machinability [51]. Thus copper pipes and copper and copper alloy fittings often contain a small amount of lead. The following sections review the standard and certification requirements for products and materials approved for use in plumbing installations to avoid excessive lead level in drinking water. The maximum allowed lead content and lead leaching limits for different products and materials should be stipulated.

#### 2.2.1. Allowable Lead Content

The maximum allowable lead content limit for different products and materials specified in the US [52,53], Canada [52,53,54,55], Australia [56,57,58,59,60], New Zealand [56,57,58,59,60], the UK [61,62,63,64,65,66,67] and Hong Kong [61,62,63,64,65,66,67] are compared in Table 2. 

Table 2 shows that the US adopts a performance-based approach [68] that requires a maximum of 0.25% weighted average lead content (*WLC*) in the entire plumbing system (the same level is used in Canada). Australia, New Zealand, the UK and Hong Kong adopt an absolute approach which sets the maximum allowed lead content in terms of % of weight of the component. However, there is no literature explaining how the limits were set.

In the US standard, Equation (1) is used for calculating the *WLC*:

(1) $$WLC=\overset{n}{\underset{i=1}{\sum }}({LC}_{i}\times \left[\frac{{WSA}_{i}}{{WSA}_{t}}\right])$$

where, *LC_i_* = maximum allowable lead content of the *i*^th^ component, % of weight; *WSA_i_* = wetted surface area of the *i*^th^ component, m^2^; *WSA_t_* = total wetted surface area of all components, m^2^; *n* = number of wetted components. 

In Australia, New Zealand, the UK and Hong Kong, the requirements set for copper pipes are more or less the same, ranging between 0.085% and 0.05% by weight, but there is a larger difference in requirements set for fittings, gate valves and faucets, ranging between 3% and 8% by weight. 

As for soldering material, the UK and Hong Kong have set an absolute lead-free requirement. The US and Canada also set an absolute requirement of 0.2% by weight. The requirement for Australia and New Zealand is 0.1% by weight.

#### 2.2.2. Leaching Limit

The leaching limit and the key test requirements specified for the US [53], Canada [53,69], Australia [70], New Zealand [70], the UK [71] and Hong Kong [72] are compared in Table 3. It is noted that the allowed leaching limits in the US and Canada are identical and the limits in Australia and New Zealand are also identical. Amongst the two groups of countries, the US and Canada set the leaching limit for a single product/material as 0.5 µg/L. The test solutions are with a minimum pH values 6.5 for pipings and 5 for fittings and valves. The sample size for immersion test has to fulfil a minimum surface area to volume ratio of 5000 mm^2^/L. The Australia/New Zealand group, with different test requirements, sets the leaching limit for a single product/material as 10 µg/L. The test solution for all products/materials is with a minimum pH value of 6.5. The sample size for immersion test has to fulfil a minimum surface area to volume ratio of 1000 mm^2^/L, and the leaching limit has to be complied with by duplicate test samples. 

For Hong Kong, the recently issued water supply booklet [72] states that all materials that come into contact with drinking water (including pipes, joints, soldering materials, valves, taps and other fittings) must comply with the relevant British Standards for potable water use. Thus it can be presumed that the lead content limit and leaching test details are identical to that of the UK. However, the UK has lead leaching test standard only for non-metallic components; there is no standard for metallic components.

#### 2.2.3. Testing and Commissioning

To verify the safety of drinking water upon completion of a plumbing installation for the protection of public health, testing of water samples is often carried out to monitor the presence of lead on both the supply side and the plumbing installations.

The US [5], Canada [42], Australia [43] and New Zealand [44] have developed their own guidance manuals for water sampling protocols. Amongst them, the US and Canada manuals are rather comprehensive, with very detailed information and guidelines while manuals in Australia and New Zealand are relatively brief. They largely refer to the World Health Organisation (WHO) Standard [4] whereby many of the requirements are cross-referenced to the International Organization for Standardization (ISO) standards [73,74,75,76] and their own regional standards [77]. 

The UK does not have its own guidance manual. The sampling requirements are set in the European Drinking Water Directive [78] which are largely based on WHO and ISO standards [4,73,74,75,76]. However, the Drinking Water Inspectorate (DWI), on behalf of the Secretary of State for Environment, Food and Rural Affairs in England and Welsh Ministers in Wales, have published a guidance manual for implementation of water supply regulations [79] which covers a little bit of water sampling protocol also.

Details of the protocols of the studied jurisdictions are compared in Table 4. The protocols include the number of drawn samples, the sampling size and locations, the container specifications and the compliance requirements. 

It can be seen that they basically adopt two tiers of samplings. Tier-1 sampling is to investigate the dissolved lead from standard plumbing fitting materials for identifying the problematic locations and implementing corrective measures. Sample collection requires a period of stagnation of a minimum of 30 min to 12 h to increase the likelihood that the concentrations of lead in the first 0.1 to 1 L of water are close to a maximum value, often called the “unflushed” sample. For the “unflushed” sample, compliance requirements are generally less stringent and require 90 to 95 percentile non-exceedance of the regulatory limit (10 µg/L to 15 µg/L) but the sampling size should be large enough to achieve 95% confidence of the population size. Sampling locations should be representative points relative to the pipe-work distribution system, which should include the dead end of distribution branches as recommended by a previous study [80]. Sampling volume is preferred to be smaller to increase the accuracy. Should the samples fail the compliance tests, Tier-2 sampling should be introduced to the high risk points.

Tier-2 sampling is to check the supply water quality to ensure 100 percentiles non-exceedance of the regulatory limit (10 µg/L to 15 µg/L). Sample collection requires a flush of the pipeline for 2 to 5 min to check the acceptability of the concentration of lead for daily use so it is often called the “flushed” sample.

In sample collection, there are different requirements on the containers to be used (treated and untreated), the allowed sample holding time, the sample delivery method, and the sampling water temperature. However, despite the differences in requirements, consensus on some basic rules still can be obtained. The consensus rules for the two tiers of sampling approach are summarised in Table 5.

## 3. Results

Based on the above, the gaps in practices followed in Hong Kong in different areas are identified as follows.

### 3.1. Governing Ordinances and Regulations

It is noted from the above that for a plumbing installation project in Hong Kong, the AP, the main contractor, and the LP are the parties/persons officially involved in ensuring safe drinking water supply to consumers. But as mentioned, the AP and the main contractor play only a limited statutory role. The LP, who is a TCP T3, is the only person to bear the statutory responsibility. Thus there is a lack of a structured quality control system for monitoring the quality of plumbing installations. 

In establishing a structured control system, reference can be made to the existing laws governing fire services installations in Hong Kong [81]. In a fire services installation, three-tier quality control system is adopted to ensure quality installation works [82]. Tier-1 requires that the plumbing installation of a fire services system be carried out by a LP to ensure installations are in compliance with regulations, design assumptions and requirements. Tier-2 requires registered Fire Services Installation Contractor responsible to carry out all fire services installations to ensure the LP’s works are monitored. Tier-3 is to rely on the AP, assisted by other registered professional engineers, who are required to sign on drawings, design calculations and completion report for submissions made to statutory authorities, to ensure design, installation and overall performance are in due compliance with relevant ordinances and regulations. 

It should be noted that a LP is needed even for plumbing installation of a fire services system which is much smaller scale than plumbing installation of a water supply system.

For satisfactory implementation of the three-tier quality control system in a plumbing installation, at the first-tier level, it is important that all workers engaged in plumbing installations must be LPs. The purpose is to ensure that works are carried out only by qualified plumbers. Thus the LPs should be appropriately qualified. There should be LPs of different grades depending on the qualifications and experience.

At the second-tier level, a registered PIC should be brought into the legislation system. All contractors engaged in plumbing installations must be PICs. The purpose is to ensure that plumbing installation work is carried out only by qualified LPs under supervision of qualified PIC. The PIC should have the competence to ensure design requirements are fulfilled, and materials used are safe. They can be hired directly by the building owner or the main contractor as nominated or a domestic contractor. 

**Figure 1:**
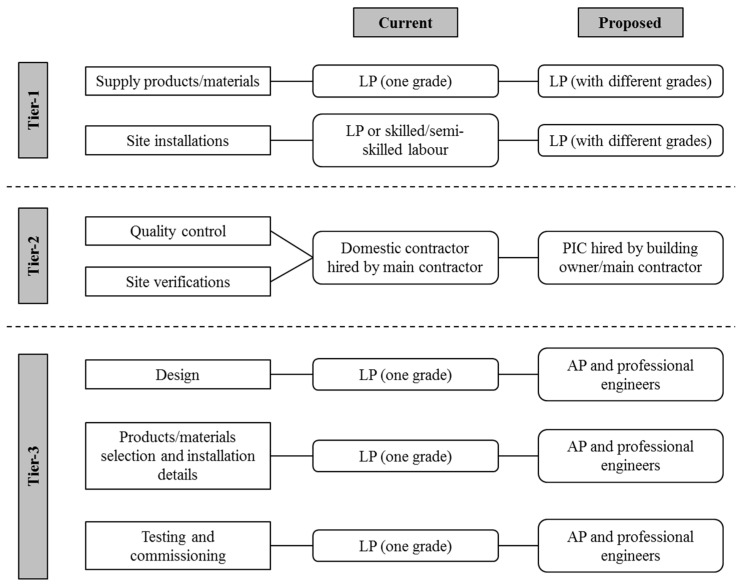
Proposed and current quality control system. Remarks: LP = licensed plumber; AP = authorized person; PIC = plumbing installation contractor.

At the third-tier level, the AP or the registered professional engineers should be made accountable for the performance of the plumbing installation and the quality of the supply water. Thus submissions of plumbing system design, materials used and installation details, currently untaken by the LP, should be done by the AP, or the registered professional engineers. The proposed 3-tier quality control system in comparison to the current system is shown in Figure 1.

### 3.2. Products and Materials

It is noted that Hong Kong follows exactly the requirements in the UK for the maximum allowable lead content in products and materials. However, considering that Hong Kong is well-known for high-rise residential buildings, plumbing installations are therefore of a much larger scale than in the UK. 

**Figure 2:**
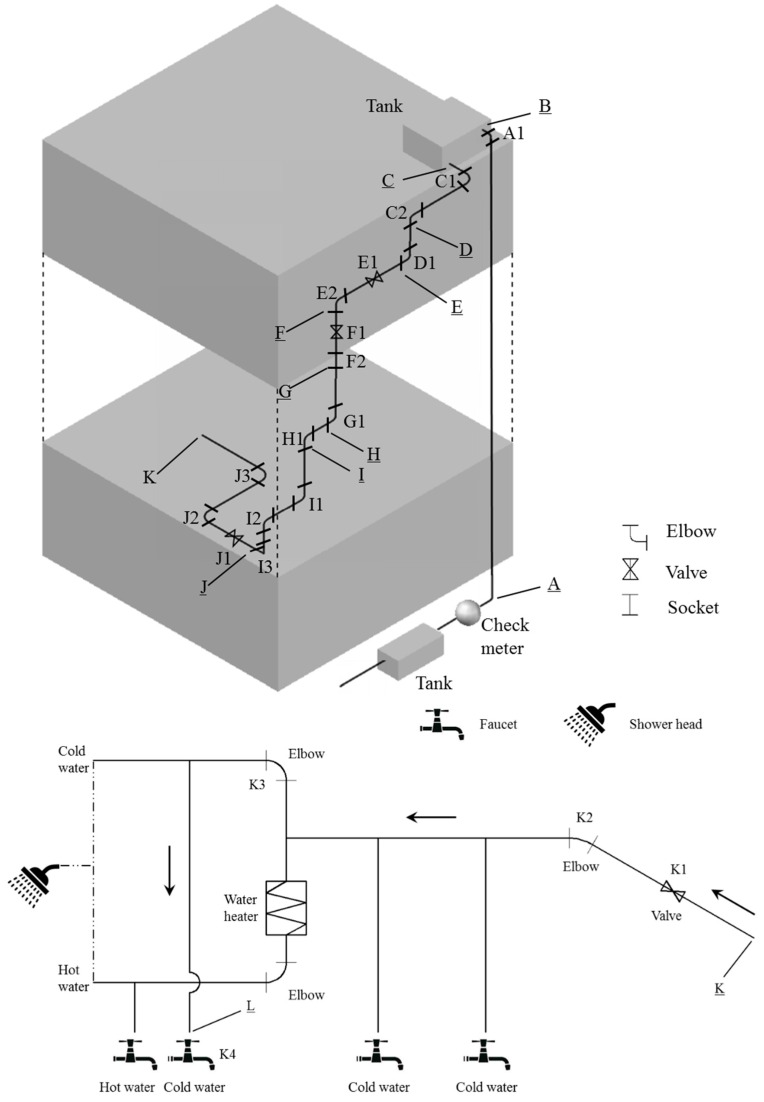
A simplified schematic diagram of the plumbing installation.

An attempt has been made to estimate the *WLC* of a plumbing installation supplying drinking water to the ground floor of a 40-storey high-rise residential building in Hong Kong. The studied building is a typical building used for investigation of the lead-in-water crisis in Hong Kong by the HKSAR government. A simplified schematic diagram of the plumbing installation is shown in Figure 2.

The *WLC* was calculated based on Equation (1). *LC_i_* was based on the UK/Hong Kong standard (Table 2), *WSA_i_* and *WSA_t_* were determined based on the actual plumbing installation. Total length of elbow/socket was assumed to be 2 times the pipe diameter whilst that of the valve/faucet are based on product information. Results are summarised in Table 6. It can be seen that the resultant *WLC* is 0.1342% which is far smaller than the performance-based requirement adopted in the US (=0.25%). 

To ascertain whether the US standard can still be fulfilled if Hong Kong follows other international standards, the *WLC* of the same installation was again calculated based on various international standards. Results are again summarized in Table 6. It can be seen that amongst the three sets of international standards, Australian/New Zealand standards are the most stringent, resulting in a *WLC* of 0.098%.

However, given that the scale of plumbing installations can have very large variations, in terms of pipe length, the number of gate valves and the associated fittings, to avoid too high a *WLC* level due to building-specific characteristics, the use of the US’s performance-based approach is recommended. 

In respect of the allowed leaching limit for products and materials, it is noted that Hong Kong does not specify the relevant requirement. To confirm whether reference can be made to other international standards, an attempt has been made to calculate if WHO’s recommended lead concentration (=10 µg/L) can be met if all products and materials used in the above-mentioned typical plumbing installation in Hong Kong (Equations (2) and Figure 2) are in compliance with single product allowable leaching (SPAL) limits of the two countries groups (0.5 µg/L for the US and Canada; and 10 µg/L for Australia and New Zealand). The calculation is based on Equations (2) and (3):

(2) $$LLC=\overset{n}{\underset{i=1}{\sum }}{(LL}_{i}\times \left[\frac{{WSA}_{i}}{{WSA}_{t}}\right])$$

and:

*LL_i_* = *SPAL* × *NF* × *N* (3)

where, *LLC* = weighted average lead leaching concentration, µg/L; *LL_i_* = maximum allowed leaching limit of the *i*th component, µg/L; *WSA_i_* = wetted surface area of the ith component, m^2^; *WSA_t_* = total wetted surface area of all components, m^2^; *n* = number of wetted components; *SPAL* = single product allowable leaching limit (Table 3), µg/L; *NF* = normalization factor; *N* = number of segments (1 for fittings).

*NF* is used to account for differences between laboratory and field surface-area-to-volume and *N* is used to account for the pipe/fitting length. For the US/Canada standards [53], a fixed *NF* is assumed for pipes of different diameters of an assumed length and N is determined based on the assumed and the actual pipe lengths. In case of Australia/New Zealand standards [70], *NF* is assumed to be a maximum of 0.1 and *N* is determined by the maximum allowed surface area to volume ratio (=15,000 mm^2^/L) and the volume of the test solution (=1 L). The calculation results are summarised in Table 7.

It can be seen in Table 7 that if all products and components leach out the maximum allowed *SPAL*, the resultant lead concentration in drinking water will be 1.76 µg/L and 293.5 µg/L for compliance with the US/Canada and Australia/New Zealand standards, respectively. The results obviously indicate that if the allowed leaching limit set by the Australia/New Zealand group, is applied to Hong Kong, the resultant lead concentration in drinking water will not be able to meet WHO’s requirement of 10 µg/L [4]. 

Furthermore, considering that lead leaching generally increases as acidity level of drinking water increases [83], pH values of drinking water in the four studied countries [42,43,84,85], together with that for the UK [79] and Hong Kong [8], are compared in Table 8. It can be seen that pH value of the test solutions specified in their test requirements (minimum 6.5 for pipings) are generally in-line with the acidity level of their drinking water (6–10.5).

Based on the acidity level of drinking water and the scale of the plumbing installations, it is evident from the above that Hong Kong has no stipulated leaching limit and leaching tests requirements should make reference to the relevant requirements adopted in the US.

### 3.3. Testing and Commissioning

Hong Kong does not have a guidance manual but it has been stated in WSD’s recent circular letter that the sampling protocol is by reference to the ISO Standards [86] which is a one-tier approach. However, the water sampling requirement in fact has never been mentioned in the relevant Ordinances and Regulations. Regarding water sampling approach, two tiers sampling protocol (Table 5) is recommended to ensure the quality of water on both the supply side and the plumbing installations.

## 4. Conclusions

To acknowledge the recommendations made for improvement in the quality control system, and the water quality standards for safeguarding the drinking water quality of new housing developments in Hong Kong, international and Hong Kong practices in terms of the governing ordinances and regulations, the products and materials used and the testing and commissioning requirements, to ensure safe drinking water supply to consumers are reviewed. The inadequacies of Hong Kong practices, which directly and indirectly lead to the lead-in-water crisis in Hong Kong, were identified. The recommended changes to the current practices can be summarised as below:

1. A 3-tier quality control system is proposed to ensure that design, installation and materials used are in accordance with requirements specified in various governing ordinances and regulations.
2. Performance-based approach should be adopted for specifying the allowable lead content in products and materials used.
3. Leaching limit and leaching test for products and materials used should be introduced to align with US requirements.
4. Two tiers sampling protocol should be adopted to ensure the quality of water on both the supply side and the plumbing installations.

The international practices reviewed and the identified gaps in practice in Hong Kong as summarized above would become useful reference for countries in strengthening their relevant water quality standards.

## Figures and Tables

**Table 1:** Comparison of legislation structure and relevant standards in various regions.

Geographic Entity	Enforcing Authority	Regulation	Standards & Guidelines	Installation
US	Environmental Protection Agency	Safe Drinking Water Act (SDWA)	Simultaneous Compliance Guidance Manual	Licensed Plumber
Canada	Health Canada	SDWA	Guidance on Controlling Corrosion in Drinking Water Distribution Systems	
Australia	Department of Health & Medical Research Council	SDWA	Australian Drinking Water Guidelines	
New Zealand	Ministry of Health	SDWA	Guidelines for Drinking-Water Quality Management for New Zealand	
UK	Secretary of State for the Environment, Transport and the Regions	Water Industry Act and Water Supply (Water Quality) Regulations	Guidance on the Implementation of the Water Supply (Water Quality) Regulations	
Hong Kong	Water Supplies Department	Waterworks Ordinance and the Sub-Legislature Waterworks Regulations	Plumbing Installation for Buildings Specific to Hong Kong	

**Table 2:** Comparison of maximum allowable lead content (by % of weight) in copper pipe and copper alloy valves and fittings in various geographic entities.

Geographic Entity	US *	Canada *	Australia	New Zealand	UK	Hong Kong
Copper Pipes	0.25%	0.25%	0.05%		0.085%	
Copper Alloy Gate Valves	0.25%	0.25%	4.5%		8%	
Facuets	0.25%	0.25%	4.5%		not specified	
Solder	0.2%	0.2%	0.1%		0%	
Other Copper Alloy Valves and Fittings	0.25%	0.25%	4.5%		3%	
Weighted-Average	≤0.25%	≤0.25%	Nil		Nil	

Remarks: * In total wetted parts.

**Table 3:** Leaching limits and leaching test requirements.

Description		US	Canada	Australia	New Zealand	UK
Test Sample		Materials or Finished Products				Only for Non-Metallic Products
Test Method		in-the-Product (all Samples except Solder); Immersion (Solder)				
Sample Size	in-the Product	Smallest Inner Diameter				
	Immersion (Minimum Surface Area to Volume Ratio)	5000 mm^2^/L		1000 mm^2^/L		
Sample Pre-Test	Cleaning	Rinsed with Contaminate-Free Water		Rinsed in Flowing Tap Water		
	Conditioning in Test Water	for Minimum 2 days		for 24 h		
Test Water	pH value	Minimum 6.5 (Pipings) and 5 (Valves and Fittings)		Minimum 6.5		
	Temperature	23 ± 2 °C		20 ± 2 °C		
Exposure Time		24 ± 1 h		24 ± 2 h		
Container	in-the Product	Capped with Inert Materials				
	Immersion Test	Materials Inert to the Test Water		Glass or Polyethylene Ware		
Single Product Allowable Leaching Limit		≤0.5 µg/L		≤10 µg/L (Duplicate Samples)		

**Table 4:** Sampling protocols for copper pipelines of different regions.

Description		US	Canada ^1^	Australia	New Zealand	UK ^2^	Hong Kong
Number of Draws		Two-Tier					One-Tier
Drawing Method	Unflushed	after a Stagnation Period of 6–12 h				30 min	not Specified
	Flushed	Flush for a While to 5 min					Flush for 2–5 min
Sample Volume (L)		1	1	0.1	0.15	1	0.25
Sampling Location		Random	Drinking or Cooking Taps	Representative Points		Consumer Taps	Determined by WSD
Sampling Size Confidence Level		95%	not Specified	95%		not Specified	Exact Number of Sampling Points Specified
Container		Fully Filled	not Specified	Thoroughly Rinsed			
Sample Holding Time		not Specified		28 Days	within the Same Day	7 Days	within the Same Day
Sampling Water Temperature		Cold	Cold	Constant	Cold	Constant	Cold
Compliance	unflushed	90%		90%	95%	100%	not Specified
		≤15 µg/L		≤10 µg/L	≤10 µg/L	≤10 µg/L	
	flushed	100%	100%				
		≤15 µg/L	≤10 µg/L				

Remarks: ^1^ Canada’s regulations are provincial. The actual sampling protocol may differ by provinces. ^2^ UK adopts Random Daytime Sampling approach to estimate the lead problem in a water supply zone. The consumer tap being sampled is not flushed before taking the water sample. Should excessive lead components be present, two-tier sampling protocol at the consumer tap will be adopted. WSD = Water Supplies Department.

**Table 5:** Consensus rules for the two tiers of sampling approach.

Description	Unflushed	Flushed
Drawing Method	after a Period of Stagnation of 30 min to 12 h	Flush at a Uniform Rate for 2 to 5 min
Sample Volume	0.1 L	
Sample Location	Sample Sites throughout the System and High Risk Areas	High Risk Areas
Sample Size	95% Confidence Level Related to Population Size	10% of the Tier-1 Sample Sites including the Problematic Sites
Compliance	95% Non-Exceedance	100% Non-Exceedance
Container	thoroughly Rinsed Container Provided by Recognized Laboratory	
Sample Holding Time	Deliver to Recognized Laboratory within a Day	
Sampling Water Temperature	Cold Water Pipeline at Constant Temperature	
Compliance	10 µg/L	

**Table 6:** Weighted lead content (*WLC*) prediction.

Item	Diameter	Pipe/Fitting	Length (m)	Wetted Surface Area (m^2^)	Ratio Wetted Surface Area	UK/Hong Kong		Australia/New Zealand		US/Canada	
						% Lead Content	% Lead Contribution	% Lead Content	% Lead Contribution	% Lead Content	% Lead Contribution
AB	150	Pipe	152	71,628.5	0.5421	0.085	0.0461	0.05	0.0271	0.25	0.1355
A1	150	Elbow	0.267	125.8	0.0010	3	0.0029	4.5	0.0043	0.25	0.0002
CD	150	Pipe	15	7068.6	0.0535	0.085	0.0045	0.05	0.0027	0.25	0.0134
C1	150	Elbow	0.3	141.4	0.0011	3	0.0032	4.5	0.0048	0.25	0.0003
C2	150	Elbow	0.3	141.4	0.0011	3	0.0032	4.5	0.0048	0.25	0.0003
DE	150	Pipe	4	1885.0	0.0143	0.085	0.0012	0.05	0.0007	0.25	0.0036
D1	150	Valve	0.267	125.8	0.0010	8	0.0076	4.5	0.0043	0.25	0.0002
EF	150	Pipe	10	4712.4	0.0357	0.085	0.0030	0.05	0.0018	0.25	0.0089
E1	150	Valve	0.267	125.8	0.0010	8	0.0076	4.5	0.0043	0.25	0.0002
E2	150	Elbow	0.3	141.4	0.0011	3	0.0032	4.5	0.0048	0.25	0.0003
FG	150	Pipe	38	17,907.1	0.1355	0.085	0.0115	0.05	0.0068	0.25	0.0339
F1	150	Valve	0.267	125.8	0.0010	8	0.0076	4.5	0.0043	0.25	0.0002
F2	150	Socket	0.267	125.8	0.0010	3	0.0029	4.5	0.0043	0.25	0.0002
GH	100	Pipe	38	11,938.1	0.0904	0.085	0.0077	0.05	0.0045	0.25	0.0226
G1	100	Elbow	0.2	62.8	0.0005	3	0.0014	4.5	0.0021	0.25	0.0001
HI	100	Pipe	38	11,938.1	0.0904	0.085	0.0077	0.05	0.0045	0.25	0.0226
H1	100	Elbow	0.2	62.8	0.0005	3	0.0014	4.5	0.0021	0.25	0.0001
IJ	100	Pipe	4	1256.6	0.0095	0.085	0.0008	0.05	0.0005	0.25	0.0024
I1	100	Elbow	0.2	62.8	0.0005	3	0.0014	4.5	0.0021	0.25	0.0001
I2	100	Valve	0.203	63.8	0.0005	8	0.0039	4.5	0.0022	0.25	0.0001
I3	100	Socket	0.203	63.8	0.0005	3	0.0014	4.5	0.0022	0.25	0.0001
JK	25	Pipe	15	1178.1	0.0089	0.085	0.0008	0.05	0.0004	0.25	0.0022
J1	25	Valve	0.165	13.0	0.0001	8	0.0008	4.5	0.0004	0.25	0.0000
J2	25	Elbow	0.05	3.9	0.0000	3	0.0001	4.5	0.0001	0.25	0.0000
J3	25	Elbow	0.05	3.9	0.0000	3	0.0001	4.5	0.0001	0.25	0.0000
KL	25	Pipe	15	1178.1	0.0089	0.085	0.0008	0.05	0.0004	0.25	0.0022
K1	25	Valve	0.165	13.0	0.0001	8	0.0008	4.5	0.0004	0.25	0.0000
K2	25	Elbow	0.05	3.9	0.0000	3	0.0001	4.5	0.0001	0.25	0.0000
K3	25	Elbow	0.05	3.9	0.0000	3	0.0001	4.5	0.0001	0.25	0.0000
K4	25	Cold water faucet	0.165	13.0	0.0001	4.5	0.0004	4.5	0.0004	0.25	0.0000
Misc	87	Solder	0.058	15.9	0.0001	0	0	0.1	0.0000	0.2	0.0000
*WLC* (%)						0.1342		0.098		0.25	

Remarks: solder joints of 2 mm wide were assumed before and after each fitting; calculations were based on Equation (1), and data on Table 2.

**Table 7:** Lead leaching concentration (*LLC*) prediction.

Item	Diameter	Pipe/Fitting	Length (m)	Wetted Surface Area (m^2^)	Ratio Wetted Surface Area	US/Canada			Australia/New Zealand		
						*N* × *NF*	*LL_i_*	*LL_i_* × Ratio Wetted Surface Area	*N* × *NF*	*LL_i_*	*LL_i_* × Ratio Wetted Surface Area
AB	150	Pipe	152	71,628.5	0.5421	4.18	2.09	1.1330	47.752	47,752.3	258.868
A1	150	Elbow	0.267	125.8	0.0010	1	0.50	0.0005	0.084	83.9	0.001
CD	150	Pipe	15	7068.6	0.0535	0.41	0.21	0.0110	4.712	4712.4	2.521
C1	150	Elbow	0.3	141.4	0.0011	1	0.50	0.0005	0.094	94.2	0.001
C2	150	Elbow	0.3	141.4	0.0011	1	0.50	0.0005	0.094	94.2	0.001
DE	150	Pipe	4	1885.0	0.0143	0.11	0.06	0.0008	1.257	1256.6	0.179
D1	150	Valve	0.267	125.8	0.0010	1	0.50	0.0005	0.084	83.9	0.001
EF	150	Pipe	10	4712.4	0.0357	0.28	0.14	0.0049	3.142	3141.6	1.120
E1	150	Valve	0.267	125.8	0.0010	1	0.50	0.0005	0.084	83.9	0.001
E2	150	Elbow	0.3	141.4	0.0011	1	0.50	0.0005	0.094	94.2	0.001
FG	150	Pipe	38	17,907.1	0.1355	1.05	0.52	0.0708	11.938	11,938.1	16.179
F1	150	Valve	0.267	125.8	0.0010	1	0.50	0.0005	0.084	83.9	0.001
F2	150	Socket	0.267	125.8	0.0010	1	0.50	0.0005	0.084	83.9	0.001
GH	100	Pipe	38	11,938.1	0.0904	1.05	0.52	0.0472	7.959	7958.7	7.191
G1	100	Elbow	0.2	62.8	0.0005	1	0.50	0.0002	0.042	41.9	2.0 × 10^−4^
HI	100	Pipe	38	11,938.1	0.0904	10.64	5.32	0.4808	7.959	7958.7	7.191
H1	100	Elbow	0.2	62.8	0.0005	1	0.50	2.4 × 10^−4^	0.042	41.9	2.0 × 10^−4^
IJ	100	Pipe	4	1256.6	0.0095	0.11	0.06	5.2 × 10^−4^	0.838	837.8	8.0 × 10^−2^
I1	100	Elbow	0.2	62.8	0.0005	1	0.50	2.4 × 10^−4^	0.042	41.9	2.0 × 10^−4^
I2	100	Valve	0.203	63.8	0.0005	1	0.50	2.4 × 10^−4^	0.043	42.5	2.1 × 10^−4^
I3	100	Socket	0.203	63.8	0.0005	1	0.50	2.4 × 10^−4^	0.043	42.5	2.1 × 10^−4^
JK	25	Pipe	15	1178.1	0.0089	0.08	0.04	3.7 × 10^−4^	0.785	785.4	7.0 × 10^−2^
J1	25	Valve	0.165	13.0	0.0001	1	0.50	4.9 × 10^−5^	0.009	8.6	8.5 × 10^−6^
J2	25	Elbow	0.05	3.9	0.0000	1	0.50	1.5 × 10^−5^	0.003	2.6	7.8 × 10^−7^
J3	25	Elbow	0.05	3.9	0.0000	1	0.50	1.5 × 10^−5^	0.003	2.6	7.8 × 10^−7^
KL	25	Pipe	15	1178.1	0.0089	0.41	0.21	1.8 × 10^−3^	0.785	785.4	0.070
K1	25	Valve	0.165	13.0	0.0001	1	0.50	4.9 × 10^−5^	0.009	8.6	8.5 × 10^−6^
K2	25	Elbow	0.05	3.9	0.0000	1	0.50	1.5 × 10^−5^	0.003	2.6	7.8 × 10^−7^
K3	25	Elbow	0.05	3.9	0.0000	1	0.50	1.5 × 10^−5^	0.003	2.6	7.8 × 10^−7^
K4	25	Cold Water Faucet	0.165	13.0	0.0001	1	0.50	4.9 × 10^−5^	0.009	8.6	8.5 × 10^−6^
Misc	87	Solder	0.058	15.9	0.0001	1	0.50	6.0 × 10^−5^	0.011	10.6	1.3 × 10^−5^
*LCC* (ug/L)								1.76	-		293.5

Remarks: *LL_i_* was estimated based on Equation (3).

**Table 8:** Drinking water pH value.

Description	US	Canada	Australia	New Zealand	UK	Hong Kong
pH Value	6.5–8.5	7–10.5	6.5–7.5	6–8	6.5–9.5	6.4–9.2
Reference	[84]	[42]	[43]	[85]	[79]	[8]
